# Surgical decompression improves recovery from neurological deficit and may provide a survival benefit in patients with diffuse large B-cell lymphoma-associated spinal cord compression: a case-series study

**DOI:** 10.1186/1477-7819-11-90

**Published:** 2013-04-19

**Authors:** Ching-Ming Chang, Hung-Chieh Chen, Youngsen Yang, Ren-Ching Wang, Wen-Li Hwang, Chieh-Lin Jerry Teng

**Affiliations:** 1 Division of Hematology/Medical Oncology, Department of Medicine, Taichung Veterans General Hospital, Taiwan 160, Section 3, Chungkang Road, Taichung 40705, Taiwan; 2 Department of Radiology, Taichung Veterans General Hospital, Taichung, Taiwan 160, Section 3, Chungkang Road, Taichung 40705, Taiwan; 3 Department of Pathology, Taichung Veterans General Hospital, Taichung, Taiwan 160, Section 3, Chungkang Road, Taichung 40705, Taiwan; 4 Department of Life Science, Tunghai University, Taichung, Taiwan 1727, Section 4, Taiwan Boulevard, Taichung 40704, Taiwan; 5 Department of Medicine, Chung Shan Medical University, Taiwan 110, Section 1, Jianguo N. Road, Taichung 40201, Taiwan

**Keywords:** Spinal cord compression, Diffuse large B-cell lymphoma, Decompression surgery

## Abstract

**Background:**

Malignancy-associated spinal cord compression is generally treated by surgical decompression, radiotherapy or a combination of both. Since diffuse large B-cell lymphoma (DLBCL) is highly sensitive to both chemotherapy and radiotherapy, the role of surgical decompression in the treatment of DLBCL-associated spinal cord compression remains unclear. We therefore conducted a retrospective review to investigate the impact of surgical decompression on recovery from neurological deficit caused by DLBCL-associated spinal cord compression and patients’ overall survival.

**Methods:**

Between March 2001 and September 2011, 497 newly diagnosed DLBCL patients were reviewed, and 11 cases had DLBCL-associated spinal cord compression. Six cases were treated surgically and five cases nonsurgically.

**Results:**

The rates of complete recovery from neurological deficit were 100% (6/6) and 20% (1/5) for patients in the surgical and nonsurgical groups, respectively (*P* = 0.015), while the median survival for patients in the surgical and nonsurgical groups was 48.6 months and 17.8 months, respectively (*P* = 0.177).

**Conclusions:**

We conclude that surgical decompression can improve recovery from neurological deficit in patients with DLBCL-associated spinal cord compression, possibly providing these patients a survival benefit.

## Background

Malignancy-associated spinal cord compression (MASC) is a serious oncological emergency of which breast cancer, prostate cancer, and lung cancer are common etiologies [1]. In 60 to 80% of MASC patients, the thoracic spine is involved [2]. Without immediate and appropriate treatment, MASC can result in irreversible neurological deficits including motor and sensory dysfunction, and urine and stool incontinence. Surgery, radiotherapy, or combinations of both are treatment options for MASC. Although surgery followed by palliative radiotherapy can release the compression immediately and facilitate mechanical stabilization of the spine directly [3], it is performed in only 10 to 15% of MASC patients. The patients’ poor performance status, delayed diagnosis, and the risks of the surgery itself may be reasons why few patients are treated with surgery alone or in combination. Rather, radiotherapy alone is the most common treatment for MASC [1]. Chemotherapy has a limited role in general in MASC treatment because tumor response usually takes a few weeks, which is too slow to avoid permanent neurological damage.

Unlike solid malignancies, diffuse large B-cell lymphoma (DLBCL) is highly sensitive to chemotherapy. After the success of a trial conducted by Coiffier [4], a combination of rituximab with cyclophosphamide, doxorubicin, vincristine, and prednisolone (CHOP) has become the standard first-line therapy against DLBCL, and largely improves both treatment response and outcome in patients with this disease [5]. For those who have only localized disease or are unable to tolerate systemic chemotherapy, radiotherapy is an effective alternative treatment. As DLBCL usually responds to chemotherapy within days, chemotherapy may be able to replace decompression surgery in DLBCL patients who have MASC. However, a study conducted by Peng and colleagues showed a negative impact of decompression surgery on survival in patients with primary spinal lymphoma complicated by spinal cord compression [6]. Owing to a lack of statistical power in that study, it remains unclear whether surgical decompression can benefit DLBCL patients with MASC.

The aims of this study were to compare the rates of complete recovery from neurological deficit and overall survival time between patients who received surgical decompression and those who did not. We also compared the overall survival time between patients who experienced complete restoration of their neurological deficit and those who experienced partial restoration in order to determine whether a better neurological recovery could translate into a better overall survival.

## Methods

### Patients

A retrospective chart review of patients treated in the Taichung Veterans General Hospital, Taiwan, between March 2001 and September 2011 was performed after approval by the institution review board. A total of 497 consecutive cases of newly diagnosed DLBCL were identified. Of these 497 patients, 11 (2.21%) patients were diagnosed with MASC. The diagnosis of DLBCL in these 11 patients was reconfirmed by a pathologist according to the World Health Organization classifications proposed in 2008 [7]. Six of the patients received surgical decompression promptly after diagnosis for MASC (surgical group), and the remaining five patients did not receive surgery (nonsurgical group).

### Patient staging

The DLBCL patients with MASC were staged according to the Ann-Arbor staging system [8]. Briefly, patients were defined as stage I if they had only a single localized spinal lesion. Stage II and III diseases were defined as DLBCL with a single bony site and contiguous or closely associated lymph nodes, and with distant nodal involvement. DLBCL with diffuse or disseminated involvement of one or more extra-lymphatic organ, including those with primary sites other than the spine, as well as DLBCL with multiple spinal segment involvement were defined as stage IV.

### Neurological response evaluation

We evaluated the impairment of spinal compression according to the American Spinal Injury Association impairment scale [9]. When no motor or sensory functions are preserved in the sacral segments S4 to S5, the compression is classified as scale A. Scale B is assigned when sensory but not motor function is preserved below the neurological level and includes the sacral segments S4 to S5. Scale C is defined as when motor function is preserved below the neurological level and more than one-half of the key muscles below the neurological level have muscle grade <3, whereas scale D is when motor function is preserved below the neurological level and at least one-half of the key muscles below the neurological level have a muscle grade ≥3. Scale E refers to cases where motor and sensory functions are normal. In this study, complete neurological recovery was defined as recovery of neurological dysfunction to scale E, regardless of the initial status.

### Performance status and DLBCL treatment response evaluation

Performance status in each DLBCL patient with MASC was evaluated according to criteria established by the Eastern Cooperative Oncology Group [10]. All 11 patients included in this study received six to eight cycles of CHOP or CHOP-like regimens as the standard treatment for their underlying DLBCL. The most effective treatment response to DLBCL in each of these 11 patients was re-evaluated according to the Response Evaluation Criteria in Solid Tumors [11], and the patient was categorized as being in complete remission, partial remission, stable disease, or progressive disease.

### Statistical analysis

Comparisons of patients’ clinical parameters and overall survival times between groups were analyzed using the Mann–Whitney *U* test or Fisher’s exact test where appropriate. A difference is considered significant if *P* <0.05. All statistical analyses were carried out using SPSS software, version 11.5 (SPSS Inc., Chicago, IL, USA).

## Results

### Patient characteristics

The clinical characteristics of the 11 patients reviewed in this study are summarized in Table 1. Our cohort consisted of three men and eight women, with a mean age of 51.6 years (range: 22 to 75 years). The median follow-up of this study was 1,040 days (range: 211 to 2,885 days). The most commonly involved segment was the thoracic spine, occurring in 63.6% of cases (7/11). Lower limb weakness and back pain were the most frequent clinical presentations, occurring in seven of the 11 patients. Of the remaining four patients, two initially presented with paraplegia while two had urine retention.

**Table 1:** Characteristics of patients with diffuse large B-cell lymphoma-associated spinal cord compression

**Patient**	**Sex**	**Age (years)**	**Stage**	**Location**	**Initial S/S**	**S/S to Tx (days)**	**PS (ECOG)**	**LDH (IU/l)**	**Extra-nodal involvement >1 site**	**ASIA scale**		**IPI**	**Initial management**	**NR**	**DR**
										**Pre Tx**	**Post Tx**				
**Surgical group (*n *= 6)**															
1	F	75	1	L	Back pain	3	1	236	No	D	E	Low	DS	CR	CR
2	M	33	1	T	Paraplegia	1	2	381	No	A	E	L-I	DS	CR	CR
3	F	44	4	T	Paraplegia	2	2	437	No	A	E	H-I	DS	CR	CR
4	F	22	4	T	Urine retention	1	2	310	Yes	C	E	H-I	DS	CR	CR
5	F	40	4	T	Low limb weakness	7	2	253	Yes	C	E	High	DS	CR	PR
6	F	58	1	L	Low limb weakness	7	1	188	No	C	E	Low	DS	CR	CR
**Nonsurgical group (*n *= 5)**															
7	M	63	1	T	Back pain	4	2	237	No	C	D	L-I	CT	PR	CR
8	F	53	4	L, S	Low limb weakness	16	3	381	Yes	B	D	H-I	RT	PR	CR
9	F	73	4	T, L	Urine retention	1	3	1,021	Yes	A	E	High	CT	CR	PR
10	F	54	4	S	Low limb weakness	3	2	223	Yes	B	D	H-I	CT	PR	PR
11	M	53	4	T, S	Back pain	30	3	304	Yes	A	B	High	CT	PR	PR

*M*, male; *F*, female; *T*, thoracic spine; *L*, lumbar spine; *S*, sacral spine; *S/S*, symptoms and signs; *Tx*, treatment; *PS*, performance status; *ECOG*, Eastern Cooperative Oncology Group; *LDH*, lactate dehydrogenase; *ASIA*, American Spinal Injury Association; *IPI*, International Prognostic Index; *L-I*, low-intermediate; *H-I*, high-intermediate *DS*, decompression surgery; *CT*, chemotherapy; *RT*, radiotherapy; *NR*, neurological response; *CR*, complete response; *PR*, partial response; *DR*, disease response.

According to the Ann-Arbor staging system, four of the 11 patients were classified as having stage I DLBCL, while it was stage IV in the other seven patients. No patients were found to have stage II or III disease. Regarding the international prognostic index classification [12], seven of the 11 patients in our cohort were in a high-intermediate or high risk group, and four of them were in a low or low-intermediate risk group.

### Patients who received surgical decompression had a higher probability of achieving complete neurological recovery

To investigate whether surgical decompression could provide DLBCL patients who had MASC with improved neurological recovery, we compared the complete neurological deficit recovery rate between patients in the surgical and nonsurgical groups. The clinical characteristics of the patients in the surgical and nonsurgical groups are shown in Table 2. Briefly, the age, gender, performance status, serum lactate dehydrogenase, extra-nodal involvement, stages, and international prognostic index classification were not significantly different between the two groups.

**Table 2:** Comparison of patients’ clinical characteristics in the surgical and nonsurgical groups

**Characteristic**	**Surgical group (*n*** **= 6)**	**Nonsurgical group (*n*** **= 5)**	***P *value**
Age (years)	45.3 ± 7.7	59.2 ± 3.9	0.247^a^
Gender			0.545^b^
Male	1 (16.7%)	2 (40.0%)	
Female	5 (83.3%)	3 (60.0%)	
Time from symptoms and signs to treatment (days)	3.5 ± 1.1	10.8 ± 5.5	0.329^a^
Performance status (ECOG)			0.455^b^
< 2	2 (33.3%)	0 (0.0%)	
≥ 2	4 (66.7%)	5 (100.0%)	
Lactate dehydrogenase (IU/l)	300.8 ± 38.4	433.2 ± 149.6	0.792^a^
Extra-nodal involvement >1 site			0.242^b^
Yes	2 (33.3%)	4 (80.0%)	
No	4 (66.7%)	1 (20.0%)	
Stage			0.545^b^
1	3 (50.0%)	1 (20.0%)	
4	3 (50.0%)	4 (80.0%)	
International Prognostic Index score			0.545^b^
Low/low-intermediate	3 (50.0%)	1 (20.0%)	
High/high-intermediate	3 (50.0%)	4 (80.0%)	
Neurological deficit recovery			0.015^b^
Complete	6 (100.0%)	1 (20.0%)	
Partial	0 (0.0%)	4 (80.0%)	
Overall survival (months)	48.6 ± 13.7	17.8 ± 3.4	0.177^a^

Data presented as mean ± standard error of the mean or number (percentage). *ECOG*, Eastern Cooperative Oncology Group. ^a^Mann–Whitney U test. ^b^Fisher’s exact test.

In our study, seven of the 11 patients (63.6%) completely recovered from their neurological deficit. Further analysis showed that all patients in the surgical group (*n* = 6) achieved complete neurological deficit recovery. However, only one of the five patients (20.0%) in the nonsurgical group recovered, suggesting that DLBCL patients with MASC who received surgical decompression have a higher probability of completely restoring their neurological deficit than those who did not (*P* = 0.015) (Table 2).

### Decompression surgery and complete neurological deficit recovery might confer a survival benefit

To analyze whether surgical decompression could provide DLBCL patients with MASC a survival benefit, we compared the overall survival time in patients between the surgical and nonsurgical groups. The results showed that the overall survival time for patients in the surgical group was 48.6 ± 13.7, while in the nonsurgical group it was 17.8 ± 3.4 months (mean ± standard error). Although not statistically significant (*P* = 0.177), patients in the surgical group also demonstrated a trend towards superior overall survival when compared with those in the nonsurgical group (Table 2).

To determine whether the trend towards improved overall survival in the surgical group was due to superior recovery from the neurological deficit, we compared the overall survival time between patients in whom the neurological function was completely restored with those in whom it was only partially restored (Table 3). Our results demonstrate that the median overall survival times for patients with complete and partial neurological recovery were 43.8 ± 12.5 and 19.1 ± 4.1 months (mean ± standard error), respectively. While patients with complete recovery showed a trend toward a better overall survival time than those with partial neurological recovery, our findings were not statistically significant (*P* = 0.412).

**Table 3:** Comparison of patients’ clinical characteristics in the complete and partial neurological deficit recovery groups

**Characteristics**	**Complete neurological deficit recovery (*n*** **= 7)**	**Partial neurologic deficit recovery (*n*** **= 4)**	***P *value**
Age (years)	49.1 ± 7.5	55.8 ± 2.4	0.648^a^
Gender			0.491^b^
Male	1 (14.3%)	2 (50.0%)	
Female	6 (85.7%)	2 (50.0%)	
Time from symptoms and signs to treatment (days)	5.0 ± 1.8	13.3 ± 6.3	0.164^a^
Performance status (ECOG)			0.491^b^
< 2	2 (28.6%)	0 (0.0%)	
≥2	5 (71.4%)	4 (100.0%)	
Lactate dehydrogenase (IU/l)	297.9 ± 32.6	286.3 ± 36.2	0.788^a^
Extra-nodal involvement >1 site			0.545^b^
Yes	3 (42.9%)	3 (75.0%)	
No	4 (57.1%)	1 (25.0%)	
Stage			1.000^b^
1	3 (42.9%)	1 (25.0%)	
4	4 (57.1%)	3 (75.0%)	
International Prognostic Index score			1.000^b^
Low/low-intermediate	3 (42.9%)	1 (25.0%)	
High/high-intermediate	4 (57.1%)	3 (75.0%)	
Overall survival (months)	43.8 ± 12.5	19.1 ± 4.1	0.412^a^

Data presented as mean ± standard error of the mean or number (percentage). *ECOG*, Eastern Cooperative Oncology Group. ^a^Mann–Whitney U test. ^b^Fisher’s exact test.

## Discussion

MASC is a rare clinical scenario, occurring in only 0.1 to 3.3% of patients with non-Hodgkin’s lymphoma [13]. In our cohort, MASC was found to occur in 2.21% of patients with DLBCL. Moreover, two patients (Patients 8 and 11) in our cohort did not receive any treatment until 2 weeks after the neurological deficit occurred. Since DLBCL is a malignancy with rapid tumor proliferation, spinal cord compression may occur within weeks of the onset of MASC. Early and correct diagnosis is therefore an important issue in DLBCL patients with MASC, and magnetic resonance imaging, which has an accuracy of 95% (sensitivity 93%, specificity 97%) [14], should be performed early and correctly when DLBCL patients have any symptoms or signs associated with spinal compression.

To further understand whether surgical decompression was the best treatment for complete restoration of neurological function in DLBCL patients with MASC, we compared the complete neurological deficit recovery rate between patients who received surgical decompression and those who did not. Results showed that all of the patients who received surgical decompression had complete abrogation of their neurological dysfunction (100%, 6/6). On the contrary, only 20.0% (1/5) of the patients who did not receive surgical decompression recovered. These data suggest that even though DLBCL is highly sensitive to both chemotherapy [15] and radiotherapy [16], immediate surgical decompression was the cornerstone for improving neurological deficit in DLBCL patients with MASC. Delayed treatment for patients in the nonsurgical group in our cohort, however, could be one of the factors affecting this result. Although it was not statistically significant, patients who received surgical decompression seemed to have a shorter time from symptom onset to spinal compression treatment than those who received nonsurgical intervention (3.5 days vs. 10.8 days; Table 2). Unfortunately, this delay is unavoidable because pathological proof always takes time. In addition, the pretreatment ASIA scores in patients who did not receive surgical intervention seemed worse, which could be another possibility responsible for the superior neurological deficit recovery in patients who received immediate surgical decompression. Because one of the five patients in the nonsurgical group exhibited complete recovery, further investigation is still required to determine whether surgical decompression should be applied to all DLBCL patients with spinal cord compression.

One factor that may provide some clues for the assessment of the utility of surgical decompression is the spine instability neoplastic score proposed by the Spine Oncology Study Group. These scores were established through systemic reviews to determine which patients harbor high risk of spinal instability and are in need of surgical treatment [17]. In this classification system, six components of spinal instability – including spine location, mechanical pain, bone lesion quality, spinal alignment, vertebral body collapse, and posterolateral involvement of spinal elements – are used in the risk score calculation. Patients with a score ≥7 are considered candidates for surgical intervention. Because this scoring system was initially established for MASC resulting from solid malignancies, such as cancers of the breast, prostate, and lung, its application to DLBCL patients with spinal cord compression remains uncertain and further validation is needed. However, this question will not be answered until more studies examining DLBCL-associated spinal cord compression in larger cohorts are available.

Since patients receiving surgical decompression had a better chance to completely abrogate their neurological deficit, we further investigated whether surgical decompression could have an additional positive impact on patient survival. Our results suggest that surgical decompression may result in superior overall survival of DLBCL patients with spinal compression, even though statistical significance was lacking. This result is in contrast to the findings of a study conducted by Peng and colleagues that showed the 5-year overall survival rates for patients with primary non-Hodgkin's lymphoma of the spine with neurological compression in surgical and nonsurgical groups were 60% and 100%, respectively [6]. More well-designed clinical trials will therefore be required to clarify this controversial conclusion.

To strengthen our conclusion that surgical decompression provided patients of DLBCL-associated spinal cord compression with an improved chance of survival by eliminating the neurological deficit, we analyzed the impact of complete recovery from neurological deficit on these patients’ survival. Our data showed that even though statistical significance could not be reached, there was an obvious trend toward better overall survival in DLBCL-associated spinal cord compression patients who completely abrogated their neurological deficit. From this result, we suggested that decreases in comorbidities associated with spinal cord compression, such as cardiopulmonary dysfunction [18], poor infection control [19], and psychological problems [20], could play an important role in the superior overall survival observed in patients with DLBCL-associated spinal cord compression who received surgical decompression.

Another issue surrounding the prediction of prognosis in DLBCL-associated MASC patients was raised by this study. Currently, the International Prognosis Index – which takes into account age, disease stage, extent of extra-nodal involvement, performance status, and serum lactate dehydrogenase level [12] – is widely used for risk classification in patients with DLBCL. However, International Prognosis Index scores could not precisely predict survival in our cohort (data not shown), possibly due to an invalid staging system and performance status evaluation. According to the Ann-Arbor staging system, the disease was classified as stage IV if the spine was not the primary site. However, it was difficult to distinguish primary spinal DLBCL from DLBCL with spinal involvement but other primary sites. In addition, patients with the involvement of multiple spinal segments were also defined as stage IV, regardless of whether there was an absence of closely associated or distant lymph node involvement. According to these staging criteria, only stage I and stage IV disease were present in our cohort. Moreover, the evaluation of performance status in patients with DLBCL-associated spinal cord compression may be imprecise. According to Eastern Cooperative Oncology Group performance status evaluation criteria, a patient is classified as grade 3 if they are capable of only limited self-care, and are confined to a bed or chair for more than 50% of waking hours. However, the neurological deficit of DLBCL-associated spinal cord compression patients could cause them to be bed-ridden at diagnosis, resulting in an underestimated performance status. This underestimated performance status can further lead to an imprecise International Prognosis Index score, causing it to be inferior to the true score. To compensate for the effects of invalid staging system and performance status evaluation, a modified prognostic prediction system specific to patients with DLBCL-associated spinal cord compression should be developed.

The major limitations of this study were the small study cohort and its retrospective nature. Because of the small cohort, we were unable to validate the spine instability neoplastic score specific to DLBCL patients with MASC. We were also unable to establish a revised International Prognosis Index score to identify the risk classification in DLBCL-associated spinal cord compression patients. In addition, although six to eight cycles of CHOP or CHOP-like regimens were sequentially delivered to all the patients in our cohort as the standard treatment for DLBCL, the roles of rituximab and radiotherapy on patients in our cohort were not evaluated due to the retrospective nature of this study.

## Conclusion

In summary, our results have demonstrated that surgical decompression improves recovery from neurological deficit, and may potentially provide a survival benefit in patients with DLBCL-associated spinal cord compression. Therapeutic modalities for improving the treatment of DLBCL, such as the introduction of rituximab [21] and high-dose chemotherapy followed by autologous hematopoietic stem cell transplantation [22], may further improve the outcome of these patients. Well-designed randomized control studies with larger cohorts are urgently warranted to answer these questions.

## Consent

This study was approved by the institution’s review board, Taichung Veterans General Hospital, Taiwan (CE12032).

## Abbreviations

CHOP: Cyclophosphamide doxorubicin, vincristine, and prednisolone; DLBCL: Diffuse large B-cell lymphoma; MASC: Malignancy-associated spinal cord compression.

## Competing interests

All authors declare that they have no competing interests.

## Authors’ contributions

C-MC, W-LH and C-LJT participated in the writing of the paper and research design. H-CC participated in the reading of radiologic findings. YY participated in patient care. R-CW participated in the pathologic diagnoses. All authors read and approved the final manuscript.
